# The ecology and evolution of the monito del monte, a relict species from the southern South America temperate forests

**DOI:** 10.1002/ece3.8645

**Published:** 2022-03-01

**Authors:** Francisco E. Fontúrbel, Lida M. Franco, Francisco Bozinovic, Julian F. Quintero‐Galvis, Carlos Mejías, Guillermo C. Amico, M. Soledad Vazquez, Pablo Sabat, Juan C. Sánchez‐Hernández, David M. Watson, Pablo Saenz‐Agudelo, Roberto F. Nespolo

**Affiliations:** ^1^ 28047 Instituto de Biología Pontificia Universidad Católica de Valparaíso Valparaíso Chile; ^2^ Millennium Nucleus of Patagonian Limit of Life (LiLi) Santiago Chile; ^3^ 27950 Facultad de Ciencias Naturales y Matemáticas Universidad de Ibagué Ibagué Colombia; ^4^ Departamento de Ecología Facultad de Ciencias Biológicas Center of Applied Ecology and Sustainability (CAPES) Pontificia Universidad Católica de Chile Santiago Chile; ^5^ 28040 Instituto de Ciencias Ambientales y Evolutivas Universidad Austral de Chile Valdivia Chile; ^6^ INIBIOMA (CONICET‐Universidad Nacional del Comahue) Bariloche Argentina; ^7^ Departamento de Ciencias Ecológicas Facultad de Ciencias Universidad de Chile Santiago Chile; ^8^ 16733 Facultad de Ciencias Ambientales y Bioquímicas Universidad de Castilla‐La Mancha Toledo España; ^9^ 1109 School of Agricultural, Environmental and Veterinary Sciences Charles Sturt University Albury NSW Australia; ^10^ Millennium Institute for Integrative Biology (iBio) Santiago Chile

**Keywords:** Australidelphia, climate change, conservation, hibernation, marsupial, seed dispersal

## Abstract

The arboreal marsupial monito del monte (genus *Dromiciops*, with two recognized species) is a paradigmatic mammal. It is the sole living representative of the order Microbiotheria, the ancestor lineage of Australian marsupials. Also, this marsupial is the unique frugivorous mammal in the temperate rainforest, being the main seed disperser of several endemic plants of this ecosystem, thus acting as keystone species. *Dromiciops* is also one of the few hibernating mammals in South America, spending half of the year in a physiological dormancy where metabolism is reduced to 10% of normal levels. This capacity to reduce energy expenditure in winter contrasts with the enormous energy turnover rate they experience in spring and summer. The unique life history strategies of this living Microbiotheria, characterized by an alternation of life in the slow and fast lanes, putatively represent ancestral traits that permitted these cold‐adapted mammals to survive in this environment. Here, we describe the ecological role of this emblematic marsupial, summarizing the ecophysiology of hibernation and sociality, updated phylogeographic relationships, reproductive cycle, trophic relationships, mutualisms, conservation, and threats. This marsupial shows high densities, despite presenting slow reproductive rates, a paradox explained by the unique characteristics of its three‐dimensional habitat. We finally suggest immediate actions to protect these species that may be threatened in the near future due to habitat destruction and climate change.

## Graphical abstract

We present a review summarizing the recent advances on the biology of the enigmatic monito del monte, a relict Gondwanan mammal from southern South America.

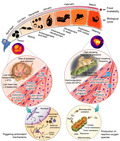

## 1 INTRODUCTION: RELICTUAL LINEAGES, MARSUPIALS, AND *DROMICIOPS*

**FIGURE 1:**
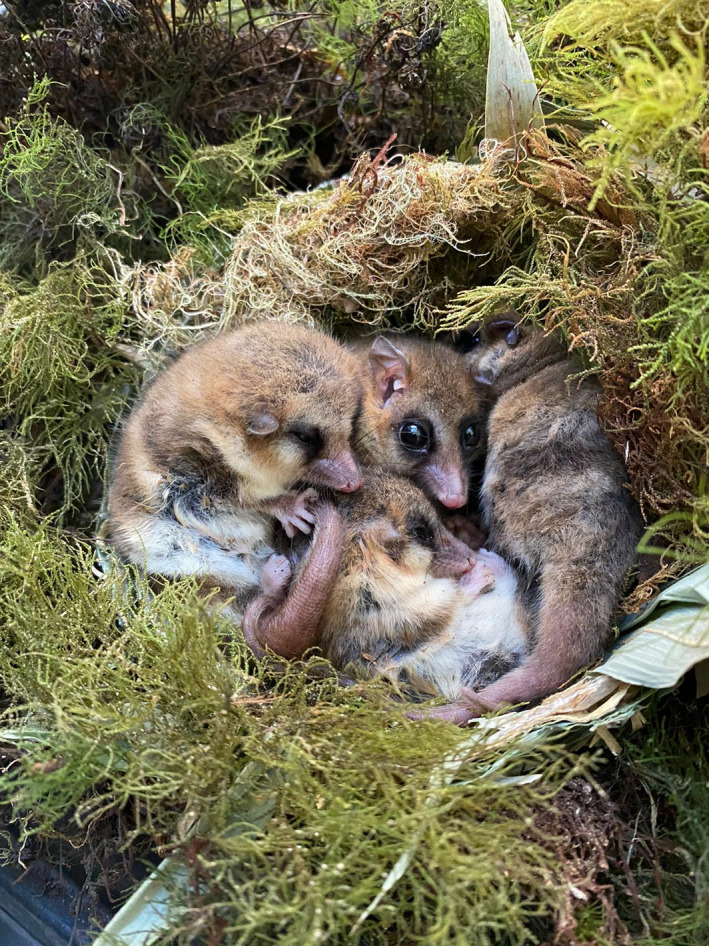
*Dromiciops gliroides* individuals found nesting together at the San Martin experimental station (Photograph: Roberto F. Nespolo)

The discovery of living representatives of groups that were thought long extinct opens a window in time to improve our understanding of their biology, as they represent invaluable material to test evolutionary hypotheses on adaptation. These relict species (sensu Habel et al., 2010) generate an enormous amount of valuable knowledge regarding ecological, morphological, and physiological traits of past lineages, as they could serve as a “window to the past” that allows us to understand the conditions that allowed them to survive for so long (Habel et al., 2010; Tan et al., 2013; Yoder et al., 2010). Here, we address one of these cases, the relict monito del monte with two recognized species: *Dromiciops gliroides* Thomas 1894 and *D*. *bozinovici* D'Elía et al. 2016 (Figure 1), an outstanding mammal from southern South America. Fossil evidence (Goin & Abello, 2013) as well as ancestral habitat reconstruction of present‐day marsupials (Mitchell et al., 2014) suggest that this marsupial (*Dromiciops*, hereafter) seems to have retained the ecological niche of its Gondwanan ancestors. In this comprehensive review, we will summarize the current knowledge on *Dromiciops* ecological role, interactions, phylogeography, physiology (with an emphasis on hibernation), reproduction, and conservation from and eco‐evolutionary perspective.

The ancestors of Marsupialia (crown‐clade Metatheria) diverged from placental mammals (crown‐clade Eutheria) at least 125 Mya in Laurasia (Bi et al., 2018; Luo et al., 2011), originating in today's China and spreading to North America, where the earliest evidence of true marsupials is known (O'Leary et al., 2013). Those early mammals remained confined to Laurasia until the late Cretaceous, when they dispersed to Gondwana, following a North America–South America path. About the same time, they suffered particularly dire consequences of the Cretaceous–Tertiary extinction in Laurasia, which ultimately drove them to extinction on the supercontinent (Case et al., 2005; Sanchez‐Villagra, 2013). In South America, marsupials thrived and diverged, eventually spreading further south and reaching Antarctica about 65–70 Mya (Mitchell et al., 2014), presumably via dispersal of Microbiotherians (Nilsson et al., 2010; Prevosti et al., 2013). This order reached Australia through Antarctica and gave origin to Australasian marsupials, which dominated the continent and adjacent islands, occupying much the same ecological niches that placental mammals did in every other continent (Long et al., 2002; Mitchell et al., 2014).

Today, marsupials are taxonomically less diverse than placental mammals, but their long and often isolated evolutionary history has resulted in a comparable morphological and ecological diversity (Sanchez‐Villagra, 2013). Extant marsupials are grouped into three American (Didelphimorphia, Microbiotheria, and Paucituberculata) and four Australasian orders (Dasyuromorphia, Diprotodontia, Notoryctemorphia, and Peramelemorphia). The evolutionary relationships among marsupial orders have long been assessed using a wide range of methods that have often yielded contradictory and intensively debated results. Particularly puzzling is the position and origin of Microbiotheria. In this regard, Szalay (1982) proposed that Microbiotheria is nested within the modern Australasian clade: Australidelphia (Meredith et al., 2008; Nilsson et al., 2010). The revolution of genetic and genomic methods during the last couple of decades has helped to disentangle the topology of the marsupial phylogeny (Eldridge et al., 2019), clearly positioning *Dromiciops* within Australidelphia, the sister group of all living Australasian marsupials (Euaustralidelphia) and confirming the monophyly of the rest of American marsupials (Duchêne et al., 2018; Mitchell et al., 2014).

### 1.1 Ecological role and interactions

Seed dispersal by Didelphid marsupials has been widely reported in tropical forests (Cáceres, 2002; Santori, De Moraes, & Cerqueira, 1995, 2004). A few marsupial species have also been involved in seed dispersal interactions in Australia (Ballardie & Whelan, 1986; Bass, 1990; Dennis, 2003) and even in New Zealand, where they are non‐native (Dungan et al., 2002; Williams et al., 2000). The high incidence of frugivory and seed dispersal on *Dromiciops* is remarkable among American and Australian marsupials, posing interesting questions about the coevolutionary processes that shaped the temperate rainforest's native flora. Recent work in Madagascar has uncovered comparable inter‐dependence between mistletoes and mouse lemurs (Cheirogaleidae). As with *Dromiciops*, these small mammals are active throughout the canopy and act as principal dispersers of mistletoe seeds in their habitats. They also undergo prolonged periods of torpor/hibernation during periods of low resource availability (Génin & Rambeloarivony, 2018, and references therein).

Mistletoes are shrubby stem parasitic plants with more than 1600 species for which dispersal represents a critical link in their life cycle (Mathiasen et al., 2008; Nickrent et al., 2010; Norton & Carpenter, 1998). Most of these plants depend on animal vectors for transporting their seeds from the parent plant to the branches of competent host plants. Mistletoes produce ripe green fruits within the South American temperate rainforests, which are not easily detected by birds (as they depend on chromatic contrast). Nevertheless, *Dromiciops* are nocturnal and locate their food primarily by scent, hearing, and vision (Amico et al., 2011), together with their capacity for color vision at the ultraviolet‐infrared spectrum (the trichromacy, discussed in the next section), permit them to be excellent foragers at night. Seed passage through *Dromiciops* digestive tract is critical for *T*. *corymbosus* germination (Amico & Aizen, 2000; Amico et al., 2017), as revealed by experimental germination trials (close to 100% of successful germination; Amico et al., 2017). Furthermore, seed establishment is strongly favored by *Dromiciops*’ climbing behavior, defecating seeds within suitable hosts and at adequate branch sizes (Amico et al., 2017), in turn impacting positively on the mistletoe regeneration rate in the forest (Amico et al., 2017; García et al., 2009). Consequently, *T*. *corymbosus* abundance and distribution is spatially correlated with *Dromiciops* presence (Fontúrbel et al., 2017a; García et al., 2009; Rodríguez‐Cabal & Branch, 2011).

The cascade of ecological services provided by *Dromiciops* extends to the whole forest community in different ways. For example, *Dromiciops* is largely responsible for the recruitment *T*. *corymbosus*, and this mistletoe represents the main nectar source for the pollinator hummingbird *Sephanoides sephaniodes* during winter (Aizen, 2003), making an indirect connection between marsupials and hummingbirds. Thus, the mutualistic relationship between the marsupial and the mistletoe may have broader ecological and evolutionary consequences at the community level. For instance, the marsupial might have allowed the mistletoe *T*. *corymbosus* to retain green coloration in mature fruits, a condition to which it is preadapted by a slower ripening process in temperate forest populations (Amico et al., 2011).

## 2 UPDATED PHYLOGEOGRAPHIC AFFINITIES WITHIN *DROMICIOPS*

**FIGURE 2:**
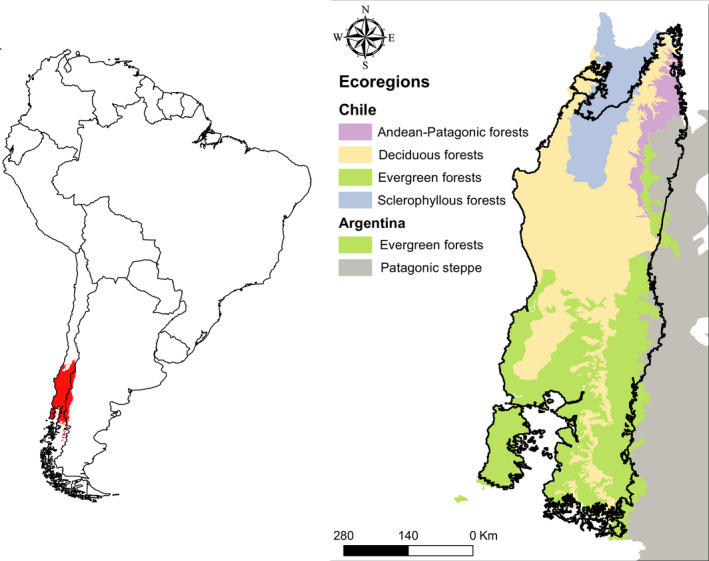
Updated *Dromiciops gliroides* distribution, including the new records extending the range to the South (based on Mejías et al., 2021; Oda et al., 2019)

*Dromiciops* distribution extends from the Chilean Pacific Coast in the west to the slopes of the Chilean Andes in the east and from the Maule Province at the north (35°S) to the Palena Province at the South (44°S) (Mejías et al., 2021; Oda et al., 2019). In Argentina, *Dromiciops* is distributed along the Andes, from Neuquén to Chubut provinces (Figure 2). A small fraction of these habitats (7%, according to Martin, 2010) corresponds to the central valley of Chile (shrubland‐type or Maulino forest habitats); while the temperate rainforest represents the remaining 93% of its distribution (Lobos et al., 2005; Martin, 2010; Saavedra & Simonetti, 2001; Uribe et al., 2017). In addition to being drier than the typical wet rainforest, the habitats occupied in the Chilean central valley receive more sunlight and have more fruits available during the summer (Fontúrbel et al., 2017a). This distribution is much smaller than the past distribution of Microbiotheria, which encompassed Bolivia, Rio de Janeiro (Middle Paleocene), Argentinean Patagonia, and the Seymour Island in Antarctica (Hershkovitz, 1999). Such distributional shrinking likely reflects major climatic changes, as the original Microbiotheria distribution (during the Miocene) was dominated by a subtropical humid climate, similar to the present‐day *Dromiciops* habitat.

**FIGURE 3:**
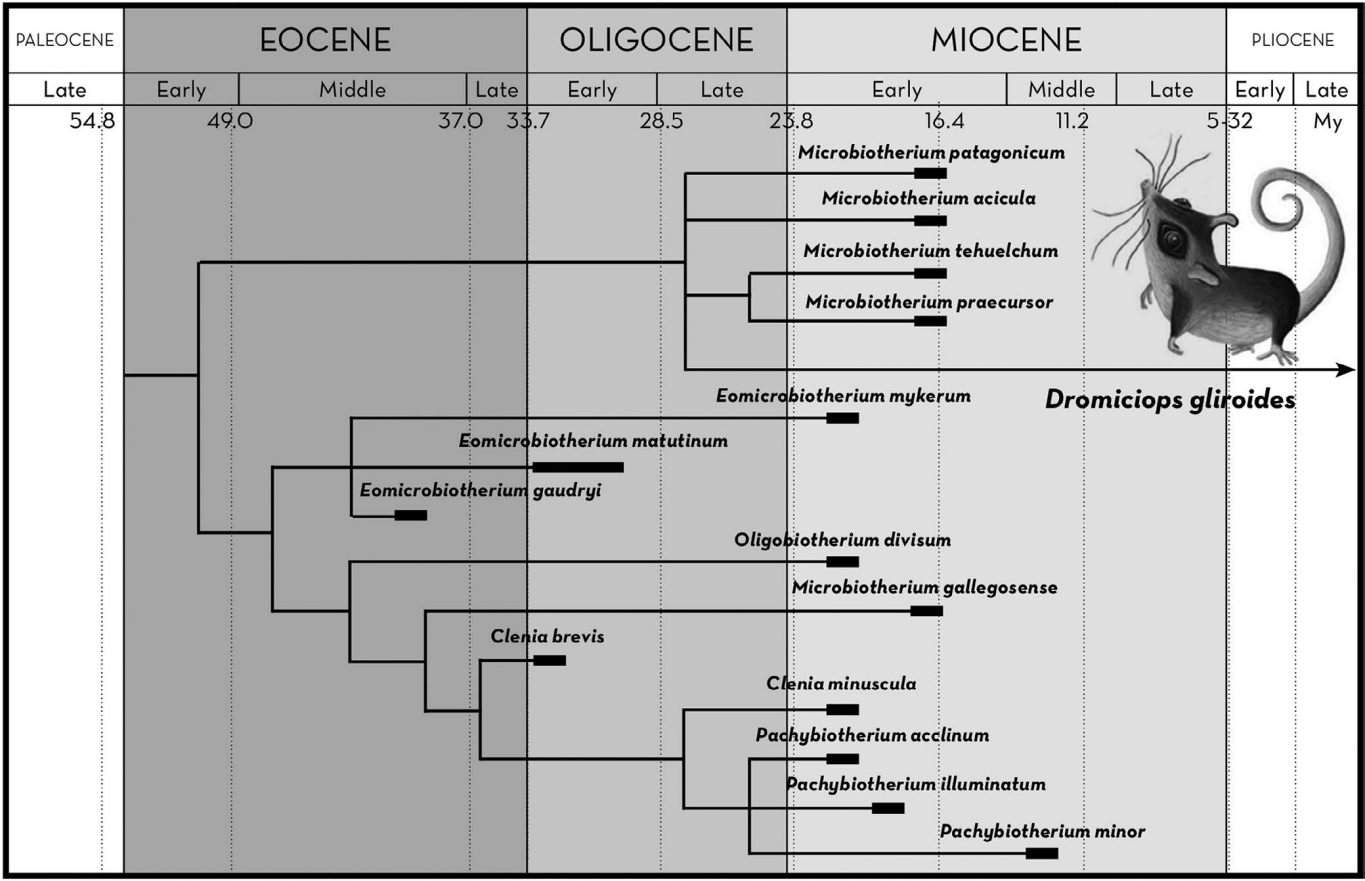
Calibrated fossil phylogeny of Microbiotheria modified from Goin and Abello (2013), showing the closeness of *D*. *gliroides* and *Microbiotherium*. According to these authors, *Dromiciops* and *Microbiotherium* are morphologically indistinguishable

The first phylogeographic analysis of *Dromiciops* populations was performed using two mitochondrial genes (Himes et al., 2008), identifying three main clades that displayed geographic structure (northern: clade “A,” central: clade “B,” and southern: clade “C” clades). Most interestingly, this study reported sequence divergence between clades A and C of 11.3%, 15.1% between A and B, and 8.2% between B and C, markedly differentiated northern and southern clades later confirmed by Valladares‐Gómez et al. (2019) using microsatellite markers. According to Himes et al. (2008), *Dromiciops* populations could have diverged in the Quaternary (1–1.8 Myr) before the last glacial maximum (~20,000 yr ago), but the deep divergence based on mitochondrial DNA suggests that these could be even older and paleontological evidence appears to agree with older divergences. Fossil evidence indicates that *Dromiciops* may be morphologically indistinguishable with *Microbiotherium*, a genus that lived between the late Oligocene and early Miocene (~29–16 Myr; Goin & Abello, 2013), which includes at least four extinct species and is considered the sister group of *Dromiciops* (Figure 3). Therefore, if *Dromiciops* is as old as these extinct lineages, this would suggest that the whole clade is as old (approximately) as the Andes mountain range (Charrier et al., 2006), and older than the scission of the Chiloé Island (ca. 10,000 years ago; Watters & Fleming, 1972). This would explain the similarities between insular and continental populations and between Argentinean and Chilean populations. The time‐calibrated phylogenetic reconstruction of these clades provided recently by Quintero‐Galvis et al. (2021) confirmed the paleontological dating of Goin and Abello (2013) and the Miocene origin of the genus.

**FIGURE 4:**
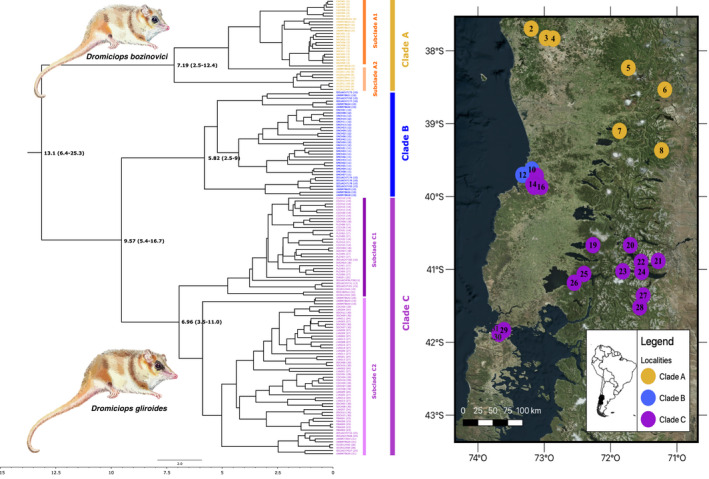
*Dromiciops* phylogenetic tree showing the support of two different species: *Dromiciops bozinovici* in the northern clade (a) and *Dromiciops gliroides* in the two southern clades (b and c). Colors show the correspondence of genetic distances and the locations in map. Illustrations of both *Dromiciops* species are shown. This figure was adapted from Quintero‐Galvis et al. (2021)

The pronounced differentiation of *Dromiciops* north–south populations together with the important morphological differentiation observed across this range have even inspired the proposition of new *Dromiciops* species (D'Elía et al., 2016). This idea, however, sparked an immediate debate based on species delimitation criteria, morphological comparisons, and genetic evidence (Martin, 2018; Valladares‐Gómez et al., 2017). Two subsequent studies (Suárez‐Villota et al., 2018; Valladares‐Gómez et al., 2019) contributed new genetic data and confirmed the existence of the “Northern” and “Southern” clusters of *Dromiciops*, but differentiation between these groups was not sufficient to warrant recognition as different species. Still, these studies covered a small percentage of this species geographic range (~1200 km). The most complete geographic sampling of *Dromiciops* populations to date was provided by Quintero‐Galvis et al. (2021), who resolved genetic distances for 31 localities covering the whole geographic range for the genus (Figure 4). Using two mtDNA and four nuclear genes, these authors proposed four clades, being the northernmost clade different enough from the other three to be considered as a different species (*Dromiciops bozinovici*). Another study by the same authors, using RAD sequencing (1856 variant SNPs), confirmed these results and proposed that the clade “C,” defined by D'Elía et al. (2016) as *D*. *mondaca*, to be redefined as a subspecies of *D*. *gliroides* (Quintero‐Galvis et al., 2022).

Phenotypically, the two *Dromiciops* species differ mainly in the fur coloration and the size of the muzzle and ears. In general, *D*. *bozinovici* has fur that is lighter in coloration and a shorter muzzle and ears than *D*. *gliroides* (see illustrations in Figure 4). Behaviorally, *D*. *bozinovici* appears to have lower activity levels and reactivity to human presence (Mejías et al., 2021). Both species are small (20–30 g, 110 mm snout–vent length) arboreal mammals, with broad carnivorous–frugivorous habits (Vazquez et al., 2018, 2021), social, sexually monomorphic, and are found on trees as tall as 30 m high in mature broadleaf forests (Godoy‐Güinao et al., 2018). Like other marsupials, they accumulate fat reserves in the body and tail, which is also prehensile. *Dromiciops* individuals are well adapted to arboreal life; they have opposable thumbs on all four limbs, exhibiting great precision and agility when they move through the canopy. They can run vertically up the bark of the *Nothofagus* at speeds of up to 1 m/s and can leap with enormous precision between distant branches up to 1 m far (Balazote‐Oliver et al., 2017; Mejías et al., 2021). This is attained by visual and cerebellar adaptations to discriminate distances in absolute darkness (di Virgilio et al., 2014; Gurovich & Ashwell, 2020), and most likely given their Australidelphia trichromate condition (color vision in the ultraviolet‐infrared spectrum; Arrese et al., 2002). The recent discovery that *Dromiciops* fur fluoresces pink with UV light supports this idea (C. Mejias, F. Goin, and R. Nespolo, personal observation using a UV lamp of 395 nm on live individuals). Such fluorescence may be useful for inter‐individual recognition in the dark (Y. Gurovich and R. Nespolo, personal observation). Thus, it is highly probable that *Dromiciops* can identify the color of the fruits (which are variable, see table 1 in Amico et al., 2009) in almost complete darkness, and in turn, detect each other with visible coloration patterns in the ultraviolet zone, as in platypuses and springhares (Anich et al., 2021; Olson et al., 2021).

## 3 THE “ALL‐PURPOSE” TROPHIC STRATEGY OF *DROMICIOPS*

The common name “monito del monte” refers to the arboreal habits of this marsupial, which includes opposable thumbs and a prehensile tail, resembling a small primate. Their agility in forest canopies permits these animals to forage efficiently on a variety of food items, for which qualitative descriptions exist based on fecal analysis and laboratory preference trials (Amico et al., 2009; Celis‐Diez et al., 2012; Cortés et al., 2011; di Virgilio et al., 2014; Meserve et al., 1988; Quijano, 2008). These studies have shown that *D*. *gliroides* does not appear to be selective (contrarily to other mammals, which select food items with specific nutrient composition, see Torres‐Contreras & Bozinovic, 1997; Woods, 2009), but rather opportunistic (i.e., dietary composition follows environmental availability, see Bozinovic et al., 2007; Cortés et al., 2011; Quijano, 2008). However, *Dromiciops* cannot fulfil its nutritional requirements only from fruits or insects. These marsupials need a mixed diet of fruits and insects to maintain a healthy body condition and a proper energy balance (Cortés et al., 2011). It is well established that differences in the digestive physiology of vertebrates reflect the historic levels of specific substrates of the natural diets, linking digestive enzyme activity, dietary flexibility, and digestive plasticity in an evolutionary context (Ramirez‐Otarola et al., 2011; Sabat et al., 1998, 1999). For instance, experiments performed in *D*. *gliroides* analyzing the expression of intestinal enzymes in response to diet acclimation revealed several‐fold changes in the activities of aminopeptidase‐N (the enzyme for processing proteins) and maltase (the enzyme for processing starch; Cortés et al., 2011). Thus, this marsupial exhibits phenotypic plasticity in the activity of intestinal enzymes, explaining its dietary flexibility (Cortés et al., 2011; Quijano, 2008).

Fruit availability represents a strong driver of *Dromiciops* dietary habits. They select individual fruits according to their size (larger fruits are preferred), exerting important selective forces on plant populations (Fontúrbel & Medel, 2017), and performing long foraging trips to disturbed forest stands to consume fleshy fruits (Amico et al., 2011; Mora & Soto‐Gamboa, 2011; Salazar & Fontúrbel, 2016; di Virgilio et al., 2014). In fact, *Dromiciops* consumes fruits from at least 16 species of shrubs, trees, vines, and particularly from the hemiparasite mistletoe *Tristerix corymbosus* (see table 1 in Amico et al., 2009).

## 4 FRUGIVORY: A RETAINED CHARACTERISTIC OF MICROBIOTHERIA?

The mutualistic relationships between *Dromiciops* and several endemic plants can be traced back in time to their ancestors. These facts furnish fascinating ideas about the trophic habits of past Microbiotheriids and their eco‐evolutionary relationships with the temperate flora of southern South America, which may have coevolved during millions of years. Highly specific and asymmetric (i.e., uneven dependence between the plant and the animal) interactions have appeared, such as the seed dispersal relationships between *Dromiciops* and the hemiparasitic mistletoe *Tristerix corymbosus* (Aizen, 2003; Amico & Aizen, 2000).

Recent work suggests an inter‐dependence between *Dromiciops* and *Tristerix*, which may also reflect an ancient association between microbiotheriids and mistletoes. Successive phylogenetic reconstructions have pushed the origin of mistletoes back further in time (Liu et al., 2018; Nickrent et al., 2010), with the growth habit now estimated to have transitioned from root parasite to aerial parasite in the Loranthaceae during the early Eocene (approximately 50 Myr ago). Based on fossil reconstructions and the modern‐day distribution of lineages on either side of this transition, it is estimated that the shift from understory up to the canopy occurred in western Gondwanaland. This time is 20 to 30 Myr prior to the origin of the modern frugivorous birds, the main dispersers of Loranthaceae mistletoes today (Liu et al., 2018). This temporal mismatch between the origins of mistletoes and their seed dispersers has been previously noted, as early Microbiotheriids are invoked as the most probable agents of mistletoe dispersal prior to the diversification of songbirds (Amico & Aizen, 2000; Restrepo et al., 2002). Recently, this idea has been taken one step further by Watson (2020), who suggested Microbiotheriids may have been indeed the selective agents responsible for the transition within Loranthaceae from root parasitic shrubs to stem parasitic mistletoes. By consuming fruits from understory shrubs and dispersing them up to the canopy, *Dromiciops* ancestors catalyzed the switch in growth habit to the upper canopy, where transitional forms likely parasitized the roots of vascular epiphytes like the present‐day *Gaiadendron* (Restrepo et al., 2002).

## 5 THE “CONSERVATIVE” REPRODUCTION OF *DROMICIOPS*

Although the basic biology of *Dromiciops* has been historically considered poorly known, this situation has drastically changed in the last decades, as several populations have been studied in detail (Balazote‐Oliver et al., 2017; Celis‐Diez et al., 2012; Fontúrbel et al., 2012; Franco et al., 2011; Kelt et al., 1999; Meserve, 1981; Meserve et al., 1988; Patterson et al., 1989). Not only inappropriate capture methods (Fontúrbel, 2010; Fontúrbel & Jiménez, 2009) but also lack of knowledge about its seasonal activity patterns led to a large underestimation of its densities (Fontúrbel & Jiménez, 2011; Franco et al., 2011; Nespolo et al., 2010). For example, captive *D*. *gliroides* individuals seem to be more active during summer than during other seasons (Aizen, 2003; Kelt & Martínez, 1989), but recently, several authors have studied *Dromiciops* activity in the field. For instance, Fontúrbel, Candia, & Botto‐Mahan et al. (2014) found that this species presents a nocturnal activity (from 19:00 h to 07:00 h), with a significant monthly variation related to resource abundance and distribution, closely related to fleshy fruit availability (di Virgilio et al., 2014; Fontúrbel et al., 2017b).

**FIGURE 5:**
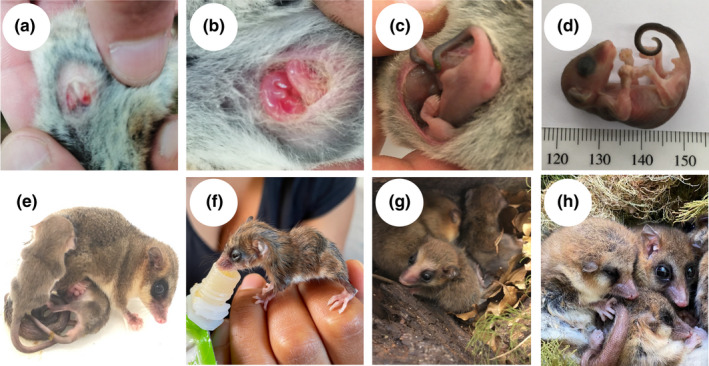
*Dromiciops* developmental stages. Panels a and b correspond to lactation in the pouch I, panels c and d correspond to lactation in the pouch II, panels e and f correspond to lactation outside the pouch, panel g correspond to juveniles, and panel h correspond to adults. Photos: R. Nespolo

**FIGURE 6:**
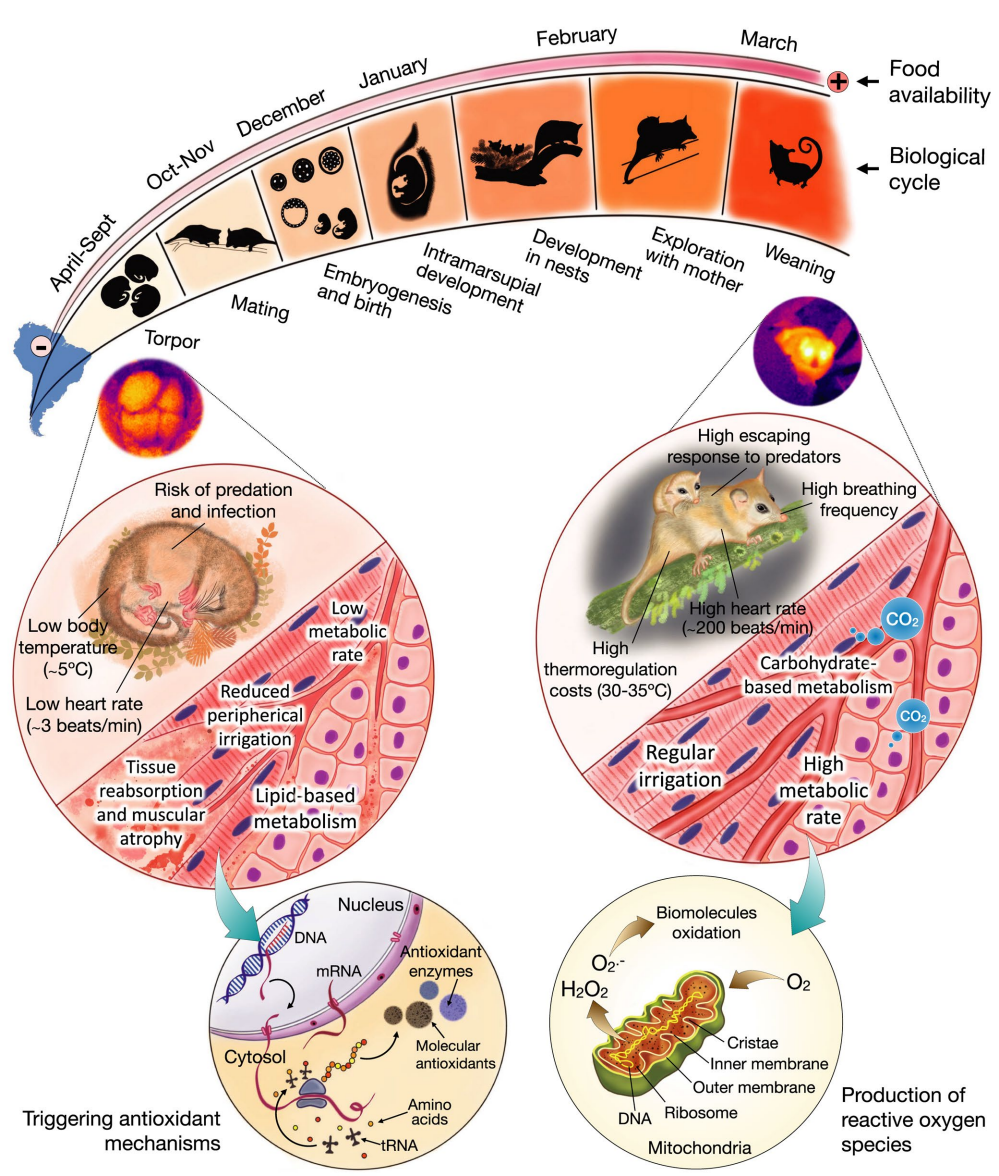
Annual cycle of *Dromiciops gliroides* showing its physiological, tissue, and biochemical changes during torpor and activity periods based on the summary provided in Nespolo et al. (2018). Particular cellular modifications involve tissue and cell protection during torpor, attained by overexpression of genes codifying for molecular chaperones and other pro‐survival mechanisms such as anti‐apoptotic pathways, mechanisms avoiding muscular atrophy and shifts from carbohydrate to fat metabolism (see details in Nespolo et al., 2018). The stages of the cycle were modified from Muñoz‐Pedreros et al. (2005), and detailed in the text (Nespolo, Fontúrbel, et al., 2022)

Historical descriptions (Hershkovitz, 1999; Mann, 1978; Muñoz‐Pedreros et al., 2005) indicate that *Dromiciops* is relatively long lived, with reports of 5‐ to 6‐year‐old individuals captured in the field (Balazote‐Oliver et al., 2017). According to Muñoz‐Pedreros et al. (2005), *D*. *gliroides* reproductive cycle is divided into seven stages. They attain sexual maturity at the second year of age and start reproducing in August–September (stage I: pair formation), then producing 1–4 pups (females have four nipples) that develop in the uterus for about a month (stage II: intra‐uterine development), and approximately in early November they migrate to the marsupium (stage III: parturition) and start lactation (stage IV: intra‐marsupium development). Several Australian marsupials are characterized by secreting milk of different compositions from different mammary glands, in close concordance to the development stage of the young (Pharo, 2019; Renfree, 1981), which is unknown for this marsupial. During the austral summer (December–January), pouched young abandon the marsupium for short exploratory excursions. However, they do not stop suckling and use the nest as a center for home‐range activities (stage V: extra‐marsupium development) (Figure 5). Finally, during February, juveniles join family excursions (stage VI: nocturnal family excursions), coinciding with the elevated trapping success usually reported. Then, juveniles become independent in March (Stage VII: juvenile independence) and start preparing for hibernation (see below). A summary of *Dromiciops* annual cycle is presented in Figure 6.

This extended *Dromiciops* breeding (i.e., 20 days of gestation and 70 days lactating), which is highly energy consuming, is combined with a low reproductive output. For instance, a female *Dromiciops* individual can produce a maximum of four offspring in a single reproductive event each year, which become fertile at the second year. This represents a maximum reproductive output of two new individuals per year (Nespolo, Saenz‐Agudelo, et al., 2022). In comparison, opossums (*Thylamys elegans*, for instance, which is sympatric with *D*. *bozinovici* in its northern distribution range) produce up to 16 individuals per reproductive event, which attain sexual maturity at the first year. This translates into a (maximum) reproductive output of 16 individuals per year. Similarly, the poorly known sympatric marsupial *Rhyncholestes raphanurus* (Caenolestidae) has been described to have continuous reproduction throughout the year, with a maximum litter size of seven young individuals (Iriarte, 2008). Reproduction in *Dromiciops* is followed by a fattening period in which animals forage frenetically to gain weight for hibernation, which starts in autumn (Nespolo, Fontúrbel, et al., 2022). The peak of energy expenditure occurs during lactation, the longest recorded in marsupials and in *Dromiciops* extends from December to January (Nespolo, Fontúrbel, et al., 2022; Nespolo, Saenz‐Agudelo, et al., 2022). To compensate for the high energy expenditure of this extravagant way of life, *Dromiciops* must reduce energy expenditure in the cold period, which is achieved by hibernation.

## 6 DAILY, SEASONAL, AND “HOT” TORPOR IN *DROMICIOPS*

The seasonal regulation of energy balance is a key concept in mammalian life histories (Harvey et al., 1991), and hibernation—a distinctive characteristic of *Dromiciops*—represents the evolution of “slow” life histories (Turbill et al., 2011). *Dromiciops* spends 6 months a year in this lethargic condition, constraining the activity period to spring and summer (Figure 6). In eutherian hibernators, there is a marked cycle of adiposity, where animals accumulate fat during summer to be consumed during hibernation, without ingesting any food during this period (Humphries et al., 2002, 2003; Toien et al., 2011). In *Dromiciops*, this cycle is unclear as they ingest food whenever they find it, which can happen even during inter‐bout arousals when hibernating (Franco et al., 2017; Nespolo, Fontúrbel, et al., 2022). In this section, we discuss distinctive aspects of seasonality and energetics of *D*. *gliroides*: its capacity for daily, seasonal torpor, and aestivation (torpor in response to hot and dry conditions).

**FIGURE 7:**
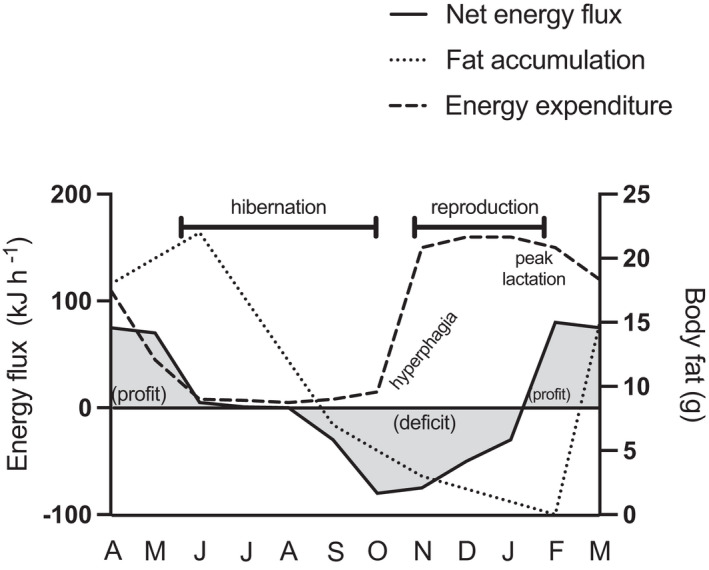
A hypothetical (but realistic) annual budget of energy and activity of a *M*_B_ = 40 g (lean mass) *Dromiciops gliroides*, summarized from descriptions of the reproductive cycle (Muñoz‐Pedreros et al., 2005), seasonal variations in activity, adiposity, and body mass (Celis‐Diez et al., 2012; Franco et al., 2017), and food availability (Franco et al., 2011; Quijano, 2008; di Virgilio et al., 2014). After reproduction, *D*. *gliroides* reduce activity and energy expenditure (dashed line) and accumulate almost twice their body size in fat (Franco et al., 2017). Fat accumulation (dotted line) was estimated from body mass fluctuations using quantitative magnetic resonance, which indicated that animals could double their body mass in autumn (Mejías et al., 2022)

Hibernation (also known as “seasonal torpor”; Geiser & Ruf, 1995) was first described in placental mammals of the northern hemisphere (e.g., squirrels, marmots, hamsters, and bears; Melvin & Andrews, 2009), where a clear pattern of seasonal metabolic depression in autumn and winter is characterized from continuous periods of activity in spring and summer (Geiser et al., 2014; Heldmaier et al., 2004). This is functionally different from daily torpor, which consists of short and shallow bouts of metabolic depression of a few hours that occur at any time of the year and is characteristic of several bat and marsupial species (Geiser, 2013; Ruf & Geiser, 2015). *Dromiciops* seems to do both, as was confirmed recently by a set of experiments under semi‐natural enclosures, indicating that in winter, animals experience seasonal torpor with multiday torpor episodes lasting 5 to 10 days, which in average represents a net energy savings of 90% compared to active individuals (Mejías et al., 2022). This complemented older studies (Bozinovic et al., 2007; Nespolo et al., 2010), indicating that *Dromiciops* experiences a dynamic form of torpor, including daily torpor of a few hours, at any moment of the year, whenever food or water is scarce (Nespolo, Fontúrbel, et al., 2022), which is considered the optimum strategy for small mammals (Bastos et al., 2021). A novel aspect of *Dromiciops* torpor was recently revealed when animals under hot torpor (also known as aestivation: metabolic depression under hot and dry conditions) were discovered in the field (Nespolo, Fontúrbel, et al., 2022). These authors described torpor in summer when temperatures were above 25°C and water was scarce. The same study described torpor in females with pups at the marsupium (pups were also in torpor, see figure 2 in Nespolo, Fontúrbel, et al., 2022). When entering into winter torpor (hibernation), animals experience a metabolic shutdown followed by passive cooling, to a limit of about –0.5°C in the tissues, and then they start thermoregulating in torpor in order to avoid freezing (Mejías et al., 2022; Nespolo, Fontúrbel, et al., 2022). The whole transition from normothermia to torpor lasts 4–6 h (Cortés et al., 2014), and happens in the nest, normally in groups of two to five individuals (Franco et al., 2011; Nespolo, Fontúrbel, et al., 2022), but arousal can be as rapid as in 30–150 min, depending on ambient temperature (Mejías et al., 2022; Nespolo, Fontúrbel, et al., 2022). These costly rewarming events are bursts of aerobic activity that could account for 25% of the energy consumed during hibernation (Mejías et al., 2022). Rewarming events during hibernation have a typical frequency in winter of about twice a month (Nespolo, Fontúrbel, et al., 2022; Nespolo et al., 2021). This probably explains the fact that monthly energy savings during hibernation (90%) are lower than the energy reduction estimated from a single torpor bout (96%, see Mejías et al., 2022). The extreme capacity to endure sub‐zero temperatures of hibernating *Dromiciops* explains its presence in high Andean locations such as Altos de Lircay at the northern edge of the distribution (Mejías et al., 2021), Llao Llao in Argentina (Rodriguez‐Cabal et al., 2008), or Futaleufú at the southern limit (Oda et al., 2019). The seasonal cycle of hibernation–activity of *Dromiciops* defines an annual energy budget with profits and loss that the animal modulates plastically. The overall balance should be positive in order to have a surplus of energy for reproduction (Figure 7).

## 7 SOCIAL THERMOREGULATION AND COMMUNAL NESTING

**FIGURE 8:**
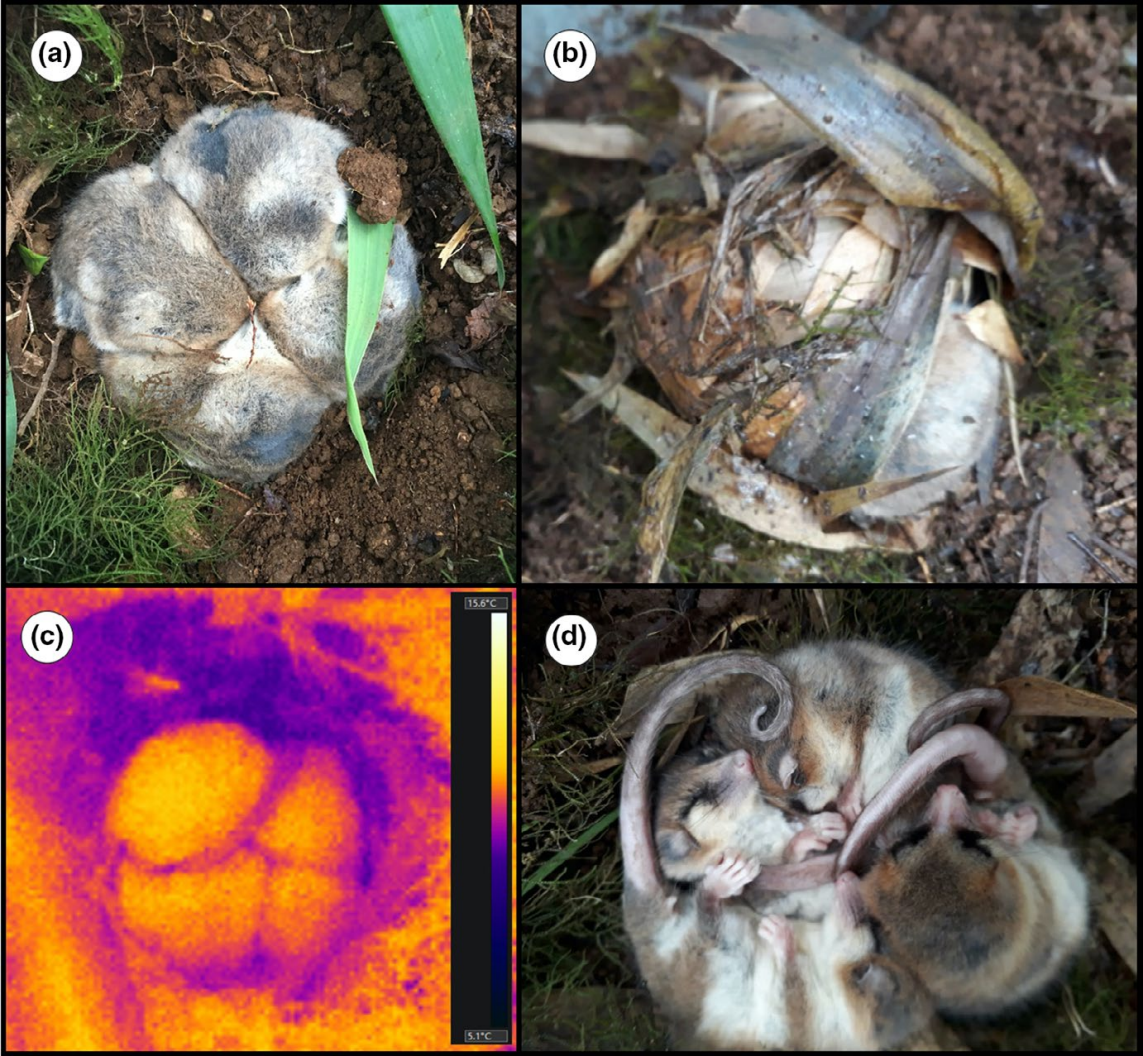
Photos of hibernating *D*. *gliroides* taken from an outdoor facility near Valdivia in May (austral autumn). (a) Four clustered individuals packed to minimize heat loss in a nest (uncovered). (b) A single individual within a typical nest built on bamboo (*Chusquea quila*) leaves and mosses. (c) A thermography of four clustered individuals showing their body temperature (~5°C, according to the color bar scale to the right). Ambient temperature is 5.2°C. (d) Lateral view of hibernating individuals. Photo credits: panel (a) R. Nespolo, panel (b) C. Mejias, panel (c) E. Oda, panel (d) P. Gutiérrez

According to Ebensperger and Labra (2020), social group living evolved under conditions where individuals obtain benefits that outweigh potential costs. These benefits could be ecological, such as reduction in predation risks thanks to alarm systems or dilution effects. The benefits could also be in terms of access to food resources due to group foraging and access to information where individuals communicate to others the location of food patches. However, thermoregulatory benefits are known to be also important for group living, especially in cold regions. This is because clustering in groups allows for better heat retention in endotherms from cold climates, thanks to the lower surface‐to‐volume area of a group, compared with a solitary individual (Canals et al., 1989). Although extensive fieldwork has shown that *Dromiciops* nests and hibernates communally (Figure 8a), as we explain in the following lines, it is unclear whether this social life is due to thermoregulatory or ecological benefits.

Nests built by *Dromiciops* are spherical, with a single entrance, built with plant materials from *Chusquea* spp. leaves, *Hymenophyllum* spp. ferns, and lined with many moss species (Figure 8b). Occasionally, nests are used more than once, although they prefer to build new ones. Based on these observations, Franco et al. (2012), Franco et al. (2011) and Celis‐Diez et al. (2012) showed that communal nesting is common in *Dromiciops* using nest boxes in two localities of southern Chile (near Valdivia and in Chiloe), with a mean of 2.3 animals per nesting box. Monitoring of nest boxes suggested that *Dromiciops* is resident throughout the year and uses torpor during cold seasons. Aging negatively correlates with *Dromiciops* communal nesting, as juveniles usually nest in groups (17% are found nesting solitary), whereas adults usually nest solitary (83% found nesting solitary; Celis‐Diez et al., 2012). Neither sex nor body mass seems to influence communal nesting in *Dromiciops*. Several Australian marsupials (Baker & Dickman, 2018), American rodents (Arnold, 1988; Boyles et al., 2008; Bustamante et al., 2002; Edelman & Koprowski, 2007; Schradin et al., 2006; Viñals et al., 2017; Wilson et al., 2010), and Neotropical bats (Roverud & Chappell, 1991) obtain significant energetic savings by hibernating in groups (Gilbert et al., 2010). However, preliminary laboratory measurements indicate that grouped individuals do not benefit from thermoregulatory savings during torpor (Franco et al., 2012). This would be suggesting that the energetic benefits of communal nesting are secondary to the benefits of sociality itself (Boix‐Hinzen & Lovegrove, 1998; Ebensperger, 2001; Schradin et al., 2006). These observations were confirmed by recent mesocosms experiments performed in hibernating individuals in the field using thermographic images, which suggest that clustered *Dromiciops* do not conserve heat better than animals hibernating in isolation (Nespolo, Fontúrbel, et al., 2022; Figure 8c). These individuals were not related, making unlikely that communal nesting is driven by kin relatedness or parental care (Franco et al., 2011; see Figure 8d). In summary, *Dromiciops* communal nesting seems to be a consequence of the advanced sociality of this species.

## 8 CONSERVATION, THREATS, AND FUTURE TRENDS

As an arboreal marsupial, a key issue for *Dromiciops* persistence is its dependence on forest habitats with certain structural features, such as tree stems, stumps, fallen logs, bamboo, and natural tree cavities (Fontúrbel, Candia, Salazar, et al., 2014). Even though the IUCN updated its threat category from Vulnerable to Near Threatened in 2011, the main problem persists: habitat loss. The southern South America temperate rainforests are rapidly being cleared due to land use change (Echeverría et al., 2006, 2007). Therefore, suitable habitat for *Dromiciops* is reduced and becoming increasingly fragmented and degraded. Habitat fragmentation has a negative effect on *Dromiciops* abundance, causing local extinction in small fragments (Rodríguez‐Cabal et al., 2007) and collapsing seed dispersal services (Amico & Aizen, 2000; Amico et al., 2011). Fragmentation threatens this marsupial as it is unable to disperse through open habitats (e.g., pastureland), remaining confined to the extant forest fragments (Fontúrbel et al., 2010).

Although some authors have recorded *Dromiciops* in exotic plantations (Fontúrbel, Candia, & Botto‐Mahan, 2014; Uribe et al., 2017), a fragment of a native forest was always found in the vicinity, where they maintain their nesting sites (Salazar & Fontúrbel, 2016). As a strict arboreal mammal, *Dromiciops* requires a dense forest with a complex three‐dimensional architecture covering the whole vertical matrix. In fact, the discovery of *Dromiciops* as high as 30 m aboveground in the canopy using camera traps was expected but difficult to document precisely (Godoy‐Güinao et al., 2018; Tejo & Fontúrbel, 2019). Forest requirements, together with frugivorous habits, configure a strong dependence on a particular ecosystem characterized by the combined presence of the native bamboo (*Chusquea* spp.) and *Nothofagus* spp. and Myrtaceae plants (Rodríguez‐Cabal & Branch, 2011), influencing the particularities of *Dromiciops* nest. Nests are considered part of an organism's “extended phenotype” (Rubalcaba et al., 2016), imprinted by the same combination of environmental and genetic factors of standard phenotypic variation. In the case of Microbiotheria, several lines of evidence suggest that the nest is fundamental for their survival (Franco et al., 2013; Hershkovitz, 1999; Honorato et al., 2016). *Dromiciops* nests are built as an oval cavity covered by a scaffold of tightly interwoven bamboo leaves combined with mosses and *Hymenophyllum* ferns (Celis‐Diez et al., 2012). This structure is impermeable and well insulated. Some authors have also attributed antimicrobial properties to it (Honorato et al., 2016), as its thick structure and the acid pH of the *Chusquea* spp. leaves may protect from predators and have a biocidal effect against parasites and pathogens.

Since *Dromiciops* distribution and abundance are influenced by several factors (particularly bamboo and mistletoe abundance; Rodríguez‐Cabal & Branch, 2011), transformed habitats can give valuable insights on *Dromiciops* persistence probabilities in a changing world. A telemetry‐based study (Salazar & Fontúrbel, 2016) showed that *D*. *gliroides* movement behavior was similar between native and transformed habitats (and consistent with other locations reported by Fontúrbel et al., 2012). However, its occurrence in transformed habitats was mainly determined by neighboring native remnants in the landscape where *Dromiciops* nests. Animals loaf in these native patches during the day, but perform foraging trips to abandoned plantations during the night (Salazar & Fontúrbel, 2016), attracted by many shade‐intolerant plant species that provide abundant fruits, such as *Aristotelia chilensis*, *Rhaphithamnus spinosus*, or *Ugni molinae* (Fontúrbel et al., 2017a).

Another major threat to this species is climate change. Given its hibernating habit, even a slight increase in winter temperatures can drastically affect them. This is because a thermal increase of 2 or 3 degrees provokes the transition from torpor to normothermia. In normothermia, animals expend 10 times more energy than in torpor, thus arousing when food is not available in winter would starve the animal to death (Nespolo et al., 2021). Thus, climate change emerges as a critical threat to this species as warmer temperatures would impose extra energy expenditure, resulting in a lower survival probability (Nespolo, Fontúrbel, et al., 2022). Furthermore, severe and prolonged droughts are a consequence of climate change in *Dromiciops*’ distribution range (Fontúrbel et al., 2021). The resulting water stress in plants causes significant reductions in flower and fruit production (Fontúrbel et al., 2018), indirectly reducing *Dromiciops* energy reserves. Altogether with the destruction of its habitats, climate change may relegate this species and, with it, the entire order to the fossil record if no actions are taken in the short term. Furthermore, *Dromiciops* local extinctions may have cascade effects in the community as a result of the loss of the seed dispersal services that this marsupial performs.

## 9 CONCLUDING REMARKS: THE ALL‐PURPOSE PHENOTYPE

According to the metabolic theory of ecology (Brown et al., 2004), populations that maintain a sustained (positive) rate of nutrient conversion into new individuals will persist compared to those that do not (Sibly & Calow, 1986; Sibly & Hone, 2002). This could be attained either by maximizing the annual reproductive rate, which in mammals is represented by small, short‐lived species (Shattuck & Williams, 2010), and in marsupials characterized by the smallest Didelphimorphia (i.e., the “fast” extreme, see Fisher et al., 2001). The “slow pace” marsupial extreme is represented by large herbivorous forms such as Vombatidae and Phalangeridae (Fisher et al., 2001). Hence, the small living Microbiotherids, with an verage reproductive output of two individuals per year (Nespolo, Saenz‐Agudelo, et al., 2022), fall in the “slow” extreme. In terms of the allometric predictions for life histories in marsupials, given by the equation: age at first reproduction =5.75*M_B_^0.10^ provided by Hamilton et al. (2011), a 30 g marsupial such as *Dromiciops* should have an age of first reproduction of 243 days (but it attains sexual maturity at 720 days). A similar computation for a maximum lifetime (=0.041*M_B_^0.20^, Hamilton et al., 2011) gives 2.5 years (but in *Dromiciops*, this parameter is above 4–5 years; Nespolo, Saenz‐Agudelo, et al., 2022). Then, the high observed densities of *Dromiciops* can only be explained by low mortality and an extended reproductive period during their lifetime, which can only be achieved in a large and spatially complex three‐dimensional habitat that serves as a refuge, such as mature temperate rainforests.

Hershkovitz (1999) proposed that Microbiotheriids’ life history is intimately associated with a combination of *Nothofagus* trees and *Chusquea* native bamboos (i.e., the *Chusquea*–*Nothofagus*–*Microbiotheria* association, CNF), which allowed them to build their sophisticated and impermeable nests that, in turn, are fundamental for hibernating in such a humid and cold forest. Thus (according to Hershkovitz, 1999), what eventually extinguished Microbiotheriids (excepting *Dromiciops*) was the disruption of the CNF by desertification at the northern edge of their distribution and freezing temperatures at the South (including Antarctica) (Hershkovitz, 1999, p10). Such phylogenetic conservatism (sensu Buckley et al., 2010) of Microbiotheriids niche is consistent with the paleontological evidence, which describes the oldest and largest Microbiotheriid known (*Woodbounodon casei*) as “a generalised non‐microbiotheriid Microbiotherian” that “resembles other frugivorous marsupials” (Goin et al., 2007).

Contemporary reconstructions also suggest that habitat preference is highly conserved across the marsupial phylogeny, as ancestral trait reconstruction of basal marsupial nodes is assigned to wet closed environments (i.e., rainforests, see figure 1 in Mitchell et al., 2014). These observations are also supported by recent evidence suggesting that mutualistic associations of Microbiotheriids with aerial mistletoes dates back to the Cretaceous (Liu et al., 2018; Watson, 2020). The ancestral marsupial that colonized Australia from Antarctica was probably little different from the present‐day Microbiotheriid, *Dromiciops*—an arboreal, nest‐building, social, omnivorous–frugivorous mammal with adaptations to the cold, seasonal, and humid canopy of the rainforest. This generalized all‐purpose animal had the potential for adapting to the new ecological niches opened by the isolation of Australia and would explain the success of colonization and posterior diversification of Australasian marsupials (F. Goin, personal communication).

## 10 FUTURE DIRECTIONS

The enigmatic biology of this relict species furnishes several exciting questions to be addressed, especially considering that abundances are extremely high in some locations (which permits to perform detailed manipulative experiments either in the lab or in the field using enclosures). Several authors already have tissue and DNA repositories in their labs, which permit developing demographic analyses and phylogeographic studies across the geographic range. Regarding the two species of *Dromiciops*, it would be interesting to characterize the potential hybrid zone existing between *D*. *bozinovici* and *D*. *gliroides* at latitude 39°S. The presence of high‐altitudinal populations also suggests local adaptation to cold conditions; thus, it would be extremely interesting to look for the physiological and molecular mechanisms for such cold adaptation. In this sense, the synchronization of the annual cycle of *Dromiciops* with the annual cycle of the forest at the altitudinal treeline is also likely, and it would be interesting to address how this coupling varies with latitudinal and altitudinal variation. In this vein, it is virtually unknown if populations are different morphologically or physiologically across the latitudinal range. Since the climatic window for reproduction is smaller at higher latitude, life history traits would be expected to vary. Would be this due to phenotypic plasticity, or is it due to genetic differences?

The enormous variation in torpor patterns of *Dromiciops* makes it an excellent model for hibernation research at the population level. Do hibernation features (e.g., duration, depth, and susceptibility to entering in torpor) target natural selection? With the great climatic variability of *Dromiciops* populations, perhaps populations at cold locations obtain comparatively higher fitness benefits than those at warm locations.

The social behavior of *Dromiciops* is also unique among marsupials, and since this species is very easy to keep in captivity, it represents a good model for experimental studies in sociality. For instance, do individuals have a permanent social structure? Do they have anti‐predator alarm systems and group warning systems for food patches? Do there exist spatial segregation during the breeding period? This list could be very long. We hope that this synthesis will help others test new hypotheses on ecology and evolution and stimulate curious minds to push the knowledge boundaries further.

## CONFLICTS OF INTEREST

The authors have no conflicts of interest to declare.

## AUTHOR CONTRIBUTIONS

**Francisco E. Fonturbel:** Conceptualization (equal); Investigation (equal); Methodology (equal); Writing – original draft (equal); Writing – review & editing (equal). **Lida M. Franco:** Conceptualization (equal); Methodology (equal); Validation (equal); Writing – review & editing (equal). **Francisco Bozinovic:** Supervision (equal); Writing – review & editing (equal). **Julian Quintero‐Galvis:** Investigation (equal); Methodology (equal); Writing – review & editing (equal). **Carlos Mejías:** Conceptualization (equal); Writing – review & editing (equal). **Guillermo C. Amico:** Conceptualization (equal); Writing – review & editing (equal). **M. Soledad Vasquez:** Data curation (equal); Validation (equal); Writing – review & editing (equal). **Pablo Sabat:** Conceptualization (equal); Writing – review & editing (equal). **Juan C. Sánchez‐Hernández:** Conceptualization (equal); Writing – review & editing (equal). **David M. Watson:** Conceptualization (equal); Writing – review & editing (equal). **Pablo Saenz‐Agudelo:** Conceptualization (equal); Writing – review & editing (equal). **Roberto F. Nespolo:** Conceptualization (equal); Funding acquisition (equal); Project administration (equal); Writing – original draft (equal).

## Data Availability

This manuscript contains no data.
